# Predictive value of chemokines (CCL 2) in bronchoalveolar lavage fluid for refractory mycoplasma pneumonia in children

**DOI:** 10.1186/s13052-023-01528-2

**Published:** 2023-09-23

**Authors:** Jiangwei Zhu, Xue Liu, Xiaowen Zhan, Mengzhu Wang, Yuling Zhang, Li Na, Shujun Li

**Affiliations:** 1 Department of Pediatrics, Huanghe Sanmenxia Hospital, Sanmenxia, 472000 China; 2 Department of Pediatrics, The First Affiliated Hospital of Xinxiang Medical University, No.88 of Jiankang road, Weihui, 453100 Henan province China

**Keywords:** Mycoplasma pneumoniae pneumonia, CCL2, Bronchoalveolar lavage fluid, Children, Refractory

## Abstract

**Background:**

There are relatively few studies investigating C-C motif chemokine ligand 2 (CCL2) level in bronchoalveolar lavage fluid (BALF) in children with Mycoplasma pneumoniae pneumonia (MPP), and the relationship between CCL2 level in BALF and refractory mycoplasma pneumoniae pneumonia (RMPP) is unclear. This study aims to explore the relationship between chemokine CCL2 level in BALF and clinical characteristics and clinical outcome in children with MPP.

**Methods:**

A total of 51 children with confirmed acute MPP and requiring bronchoalveolar lavage in Department of Pediatrics, Huanghe Sanmenxia Hospital and The First Clinical College of Xinxiang Medical University from October 2021 to February 2023 were selected as the study group. And 11 children with bronchial foreign body were selected as the control group. The study group was divided into the non-refractory mycoplasma pneumoniae pneumonia (NRMPP) group and the RMPP group based on the response to treatment. BALF and clinical data of the patients were collected. And CCL2 levels were tested in the patients. Differences in CCL2 level in BALF and clinical characteristics were tested and compared.

**Results:**

The CCL2 level in BALF of the study group was higher than that of the control group, with significant difference (P < 0.05). With ROC curve, the area under the curve (AUC) of CCL2 in BALF predicting RMPP was 0.94, the cut-off value was 0.645 ng/ml, the sensitivity was 85%, and the specificity was 94%, and the diagnostic value was better than that of serum CRP and LDH. Logistic regression analysis was used to build the RMPP prediction model, and CCL2 showed good predictive value.

**Conclusion:**

The level of CCL2 in BALF was high in children with MPP and had a high predictive value for RMPP. CCL2 can be used as one of the biomarkers for predicting RMPP.

## Introduction

Mycoplasma pneumoniae pneumonia (MPP) is more common in children and adolescents, accounting for approximately 10–40% of community-acquired pneumonia (CAP) in hospitalized children. The pathogenesis is related to mycoplasma (MP) adhesion to host cells, direct cytotoxicity to host cells, immune damage induced by inflammatory response, and immune escape [1]. The disease often presents with fever, cough, shortness of breath and other clinical manifestations [2, 3]. The condition of most patients was mild and self-limiting, but about 18% of the children need hospitalization. Without timely and effective intervention, it can develop into severe or refractory mycoplasma pneumoniae pneumonia (RMPP) [4], which can damage multiple organs and systems throughout the body, threaten the life of children or cause atelectasis, lung necrosis and other sequelae, and is an important cause of death in children under 5 years old [5].

The incidence of RMPP has increased globally, especially in Asia [6, 7], but the pathogenesis is currently unclear. Most studies believe that excessive immune response plays an important role in promoting the pathogenesis of RMPP [8]. Other studies suggest that RMPP is related to mixed infection, macrolide-resistant Mycoplasma pneumoniae (MRMP) infection, hypercoagulable blood status, mycoplasma pneumoniae load, and airway mucus plugs [4]. Early prediction and treatment can improve the outcomes. A meta-analysis conducted by Gong H and others [9] found that fever for more than 10 days, pleural effusion, extrapulmonary complications, X-ray consolidation of lung over 2/3 and CRP over 40 mg/L were risk factors for early recognition of RMPP. CRP is the most commonly used marker of non-specific inflammation in the acute phase. CRP increases when the body develops an inflammatory reaction and tissue damage caused by infection, and quickly drops to normal after remission [10]. Koster et al. showed that the level of C-reactive protein (CRP) had a high diagnostic value for pneumonia in children [11]. LDH is a non-specific inflammatory biomarker of common tissue damage and is widespread in human organs. LDH is elevated in serum when cells undergo lysis or cell membranes are damaged [12]. The results of Lu A and others [13] showed that LDH is an important indicator to predict RMPP, but the sensitivity or specificity of the individual indicator is low [14].

Chemokines are a large class of small cytokines associated with local inflammation. At present, more than 50 chemokines have been found in human body, with more than 20 kinds of chemokine receptor. Chemokines and chemokine receptors interact to constitute a complex chemokine regulatory network, which can specifically control immune cell activities and participate in immune and inflammatory responses [15, 16]. It has been shown that cytokines or chemokines may be the biomarkers for predicting the development of RMPP [17]. C-C motif chemokine ligand 2 (CCL 2) is an important pro-inflammatory chemokine, involved in inflammation, immunity, trauma and other processes [18]. Previous studies mainly focused on the chemokine CCL2 in serum. Bronchoalveolar lavage fluid (BALF) is the local liquid environment of the lung and lower airway. The biochemical and cytological changes in BALF can directly reflect the pathophysiological state of the lung. Compared with serum markers, the changes of biomarkers in BALF have higher specificity and sensitivity, and can indicate the progression of lung diseases earlier and more accurately. In an animal study, Hue Erika et al. [19] found that the cytokine levels in bronchoalveolar lavage fluid (BALF) of the affected side were significantly higher than those in the healthy side, possibly due to further lung injury caused by excessive activation of immune cells. The strong local inflammatory response of MPP suggests that the chemokines in BALF may be more meaningful for the prediction of RMPP. There are relatively few studies investigating CCL2 level in BALF in children with MPP, and the relationship between CCL2 level in BALF and RMPP is unclear. This study aims to explore the relationship between CCL2 and the clinical characteristics and disease outcome of the children to provide reference for clinical diagnosis and treatment.

## Materials and methods

### Study subjects

Children diagnosed with acute MPP and requiring bronchoscopy or treatment according to the Guideline of pediatric flexible bronchoscopy in China (2018 version) [20] in the pediatrics department and PICU of Yellow River Sanmenxia Hospital and Huanghe Sanmenxia Hospital and The First Clinical College of Xinxiang Medical University from October 2021 to February 2023 were selected as the study group. The treatment was conducted in accordance with the Guideline for diagnosis and treatment of community-acquired pneumonia in Children (2019 version) [4]. Based on the response to treatment and development of the disease, the study group was divided into the non-refractory mycoplasma pneumoniae pneumonia (NRMPP) group and the RMPP group according to the diagnostic criteria for RMPP in the Expert consensus on diagnosis and treatment of mycoplasma pneumoniae pneumonia in children (2015) [1]. In addition, 11 children with bronchial foreign bodies in the same period were selected as the control group (meeting the ethical requirements). Clinical data of 87 children with MPP were collected, including 16 cases with mixed infections, 5 cases with congenital underlying diseases, and 4 cases with incomplete clinical data. According to the inclusion and exclusion criteria, 62 study subjects (51 patients in the study group and 11 patients in the control group) were finally included. This study was approved by the Medical Ethics Committee of our hospital, with the ethics number 2,019,064, and with informed consent from the guardians.

#### Inclusion criteria and exclusion criteria

Inclusion criteria for the study group: ① Age: 1 month-14 years old (including 14 years old); ② MPP diagnosis: In this study, according to the responsiveness to treatment, MPP was divided into NRMPP and RMPP. MPP is mainly diagnosed by clinical and imaging findings [1]. The main clinical manifestations are fever and cough, which may be accompanied by headache, runny nose, sore throat and ear pain. Reduced breathing sounds and moist or dry rales can be heard in the lungs. Physical examination and chest X-ray showed pulmonary lesions, CT examination showed pneumonia [4]. In the early stage, the thickening and increase of the texture around the bronchial vessels and the thickening of the bronchial wall were seen in the imaging. The progress of the disease can be seen ground glass shadow, patchy infiltration shadow, pulmonary segment or lobe consolidation, etc., and may be combined with pleural effusion. And meet one or two of the following criteria: (1) single serum MP antibody titer ≥ 1:160 (particle agglutination method); in the course of the disease, the titer of pair serum MP antibody increased by 4 times or more. (2) MPP-DNA or RNA was positive by polymerase chain reaction. If the children diagnosed with MPP are treated with macrolide antibiotics for 7 days or longer, the body temperature continues to rise, the clinical symptoms and imaging findings are aggravated, and extrapulmonary complications occur, they will be diagnosed as RMPP. Children who were diagnosed as MPP but did not meet the RMPP diagnostic criteria would be diagnosed as NRMPP. ③ Bronchoscopy indications: lung consolidation or unexplained need treatment, in line with the relevant guidelines [20]. ④ The guardian was informed and agreed to this study.

Inclusion criteria for the control group: ① The diagnosis and treatment of tracheobronchial foreign bodies in children met the criteria of expert consensus on the diagnosis and treatment of tracheobronchial foreign bodies in children [21]. There was a history of foreign body inhalation and clinical manifestations. Physical examination and imaging examination confirmed it. ② The lavage time was within 1 week of disease onset and the co-infection was excluded. ③ The guardian was informed and agreed to this study.

Exclusion criteria: ① Incomplete clinical data; ② The patient had immune deficiency and had used immunomodulators (such as corticosteroids, cyclophosphamide, etc.) 3 months before admission; ③ Patients with bronchopulmonary dysplasia, congenital heart disease and other underlying cardiopulmonary diseases; ④ Patients with tuberculosis infection or other mixed pathogen infection, with clinically relevant manifestations of infection; ⑤ The informed consent was not obtained.

#### General clinical data

Clinical data were collected from 62 included children (51 children in the study group and 11 children in the control group), including general clinical data, such as age, gender, body weight, BMI, duration of fever, MP antibody, MP load in BALF (< 500copies/ml was negative), WBC, NE%, MONO, MONO%, PLT, CRP, LDH, D-dimer, number of lung lobes invaded and complications.

#### Pneumonia treatment plan

In this study, all the patients were treated with anti-MP according to the standard. Azithromycin was the first choice, 10 mg/(kg.d), qd, for about 7 days. After an interval of 3–4 days, if the disease needs to start the second course of treatment, the total course of treatment depends on the condition. RMPP was signed to doxycycline, levofloxacin, moxifloxacin and other treatments according to the condition and the opinions of family members. Other treatments include bronchoscopic interventional therapy, glucocorticoid therapy, intravenous immunoglobulin G (IVIG) therapy, and anticoagulant therapy.

### Main experimental instruments and experimental reagents

#### Main experimental instruments (Table 1)

**Table 1:** Main experimental instruments

Name of instrument	Brand / Company	Place of production
Fiberoptic bronchoscope	Olympus	Japan
Endoscopic washing and disinfection workstation	Beijing Wanjin Zhaoyuan Disinfection Technology Co., LTD	China
Normal temperature centrifuge	Thermo	America
Refrigerated centrifuge	Thermo	America
Purified water	Thermo	America
Multiskan Spectrum Spectrophotomete	Epoch2	America
37℃ incubator	Thermo	America
General refrigerator	Frectec	China
-80° refrigerator	Thermo	America

#### Main experimental reagents

Human MCP-1 (Monocyte Chemotactic Protein 1) ELISA Kit kit (Item No.: EH0222; Specification: 96T; Scope: 15.625-1000pg/ml), Wuhan Fei En Biotechnology Co., Ltd., China.

### Experimental methods

#### Collection of BALF and tracheal foreign body specimens

Bronchoscopy and BALF collection procedures are performed according to the Guideline of pediatric flexible bronchoscopy in China (2018 version) [20]. The removal of tracheal foreign body was carried out in accordance with Experts consensus on diagnosis and treatment of tracheobronchial foreign bodies in children [21]. With the consent of the guardian, a small amount of normal saline was used for lavage after removal, and the specimen was retained.

#### Detection of CCL2 in BALF

The Human MCP-1 (Monocyte Chemotactic Protein 1) ELISA Kit produced by Wuhan Feen Biotechnology Co Ltd was used. The operation process is carried out in strict accordance with the reagent instructions.

#### Reference range of CCL2 in children with mycoplasma pneumonia

According to the conclusion of Yang M et al., the reference range of CCL2 in the control group was 310.95 ± 45.42pg/ml, the reference range of CCL2 in the MPP group was 1820.35 ± 281.8pg/ml, and the reference range of CCL2 in the RMPP group was 3173.96 ± 377.59pg/ml [22].

### Statistical analysis

The resulting data were analyzed using the SPSS 26.0 software. Qualitative data is expressed by the rate or composition ratio (%), and comparisons between groups were performed using the chi-square test. Quantitative data were described by the mean ± standard deviation (‾x ± s) or median, interquartile range M (IQR) according to the data distribution, and comparisons between groups were performed using the independent sample T-test or the non-parametric rank-sum test. The assessment value of CCL2 level in BALF for RMPP was evaluated using the receiver operating characteristic (ROC) curve. The risk factors for RMPP were analyzed with logistic regression model. And a prediction model was established based on the results. Significance level α = 0.05.

## Results

### Comparison of clinical data and CCL2 in BALF between the study group (51 cases) and the control group (11 cases)

**Fig. 1:**
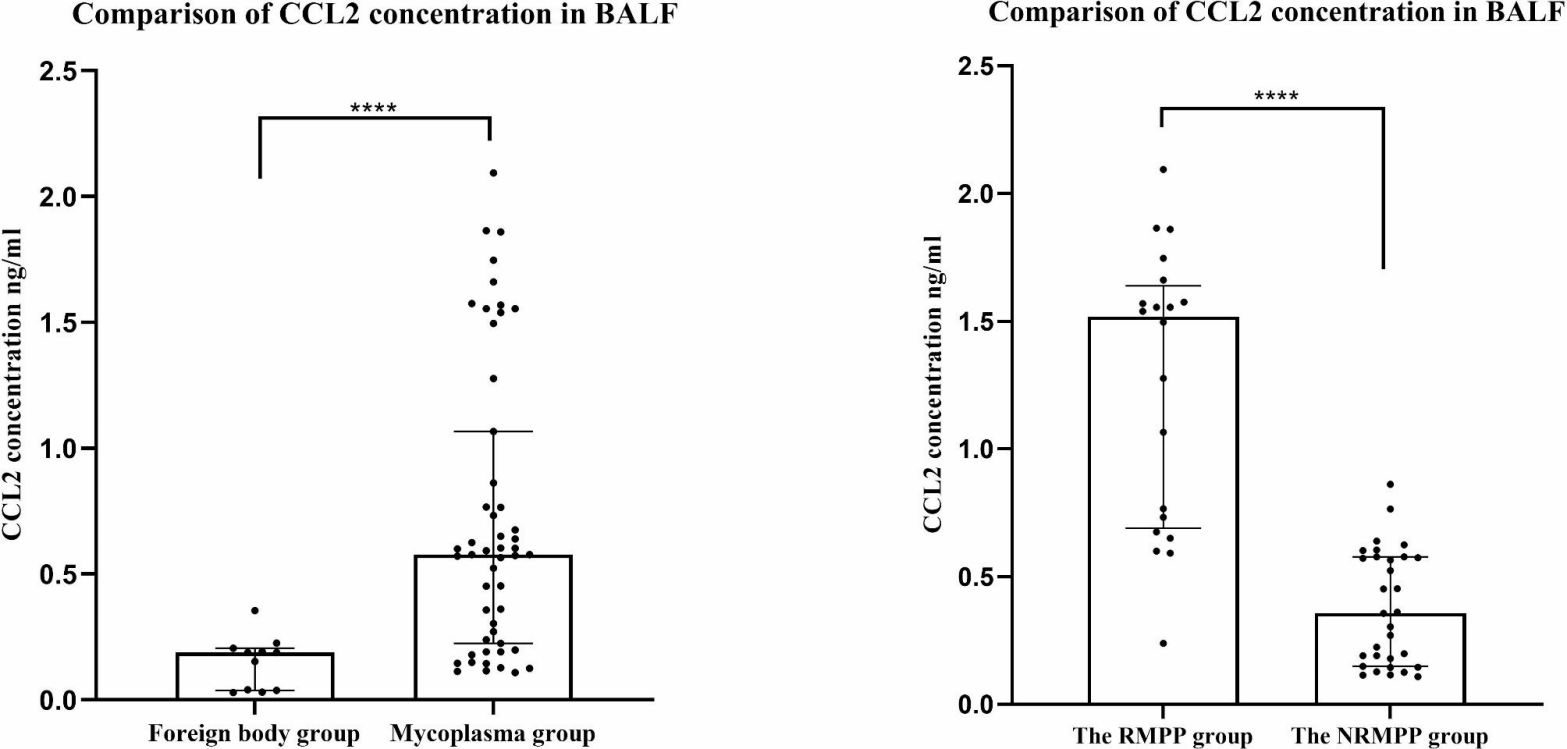
**A**. Comparison of CCL2 concentration in BALF between the study group (Mycoplasma pneumonia group) and the control group (foreign body group), indicates *P* < 0.0001 **B**. Comparison of CCL2 concentration in BALF between the RMPP group and the NRMPP group, indicates *P* < 0.0001 Note: CCL2: C-C motif chemokine ligand 2; BALF: bronchoalveolar lavage fluid; RMPP: refractory mycoplasma pneumoniae pneumonia; NRMPP: non-refractory mycoplasma pneumoniae pneumonia

Age and height of the two groups were fit with the normal distribution, and body weight and BMI did not meet the normal distribution. Genders were compared using the chi-square test, and non-parametric rank sum test was used for comparison of other items between the two groups. The study group included 22 boys (43.1%) and 29 girls (56.9%); and the control group had 4 boys (36.4%) and 7 girls (63.6%). There was no significant difference in gender and BMI between the study group and the control group (*P* > 0. 05). CCL2 level in BALF, NE%, CRP, D-dimer, and LDH in the study group were significantly higher than those in the control group, with statistically significant differences (*P* < 0.05). Table 2; Fig. 1A.

**Table 2:** Comparison of clinical data and CCL2 in BALF between the study group and the control group

Clinical characteristics	The study group(n = 51)	The control group(n = 11)	*χ* ^*2*^ */Z*	*P*
Gender (male / female)	22/29	4/7	0. 170	0. 680
Age(‾x ± s, years old)	6.2 ± 2.9	2.6 ± 2.2	-3.478	<0.001
Height(‾x ± s, cm)	117.7 ± 22.6	88.9 ± 12.8	-3.687	<0.001
Body weight M(IQR), kg	23(16, 28)	13(12, 15)	-3.301	<0.001
BMI M(IQR)	15.9(14.3, 17.7)	17.4(16.9, 18.5)	-1.760	0.079
CCL2(ng/mL)	0.578(0.224, 1.066)	0.188(0.038, 0.205)	-3.731	<0.001
NE%	69.2(77.8, 58.2)	38.5(37.3, 56.4)	-3.713	<0.001
CRP(mg/L)	20.4(10.2, 69.3)	0.36(0.20, 3.69)	-3.750	<0.001
D-dimer(ug/ml)	1.7(0.9, 4.1)	0.6(0.4, 0.6)	-3.856	<0.001
LDH(U/L)	373(265, 509)	286(234, 303)	-2.147	0.032

Note: BMI: Body Mass Index; IQR: interquartile range; CCL2: C-C motif chemokine ligand 2; NE%: neutrophil percentage; CRP: C-reactive protein; LDH: lactate dehydrogenase

### Comparison of clinical data and CCL2 in BALF between the RMPP group (20cases) and the NRMPP group (31cases)

In the RMPP group, CCL2 level in BALF, age, NE%, CRP, D-dimer, and LDH in the RMPP group were significantly higher than those in the NRMPP group, and MONO and MONO% were lower than those in the NRMPP group, with statistically significant differences (*P* < 0.05). There was no statistically significant difference in the remaining clinical data between the two groups (*P* > 0.05). There was no significant difference in the number of lobes invaded between the RMPP group and the NRMPP group with continuous variable (*P* > 0.05). Table 3; Fig. 1B.

**Table 3:** Comparison of clinical data and CCL2 in BALF between the RMPP group and the NRMPP group

Clinical characteristics	The RMPP group(n = 20)	The NRMPP group(n = 31)	*Z*	*P*
CCL2(ng/mL)	1.518(0.690, 1.639)	0.357(0.149, 0.577)	-5.267	<0.001
Age(years old)	7.0(6.1, 9.0)	5.3(3.1, 7.0)	-2.783	0.005
NE%	81.2(72.2, 87.2)	63.5(48.8, 69.6)	-4.467	<0.001
CRP(mg/L)	96.3(19.3, 133.3)	15.3(5.1, 23.2)	-3.781	<0.001
D-dimer(ug/ml)	4.35(2.35, 7.40)	1.2(0.8, 1.7)	-4.325	<0.001
LDH(U/L)	562(439, 1036)	293(231, 373)	-4.910	<0.001
MONO%	3.65(2.40, 5.58)	5.2(3.7, 7.0)	-2.239	0.025
MONO(×10^9^/L)	0.34(0.20, 0.52)	0.52(0.34, 0.63)	-2.374	0.018
Number of lung lobes invaded (lobe)	2(1, 3)	2(1, 2)	-1.478	0.139

Note: *P* < 0.05 indicates a statistically significant difference between the two groups; *P* > 0.05 indicates no statistically significant difference between the two groups; CCL2: C-C motif chemokine ligand 2; NE%: neutrophil percentage; CRP: C-reactive protein; LDH: lactate dehydrogenase; MONO%: Percentage of monocytes in white blood cells

### Risk factor analysis related to RMPP

#### ROC curve analysis of predictive value of CCL2, serum CRP and LDH in BALF for RMPP

**Fig. 2:**
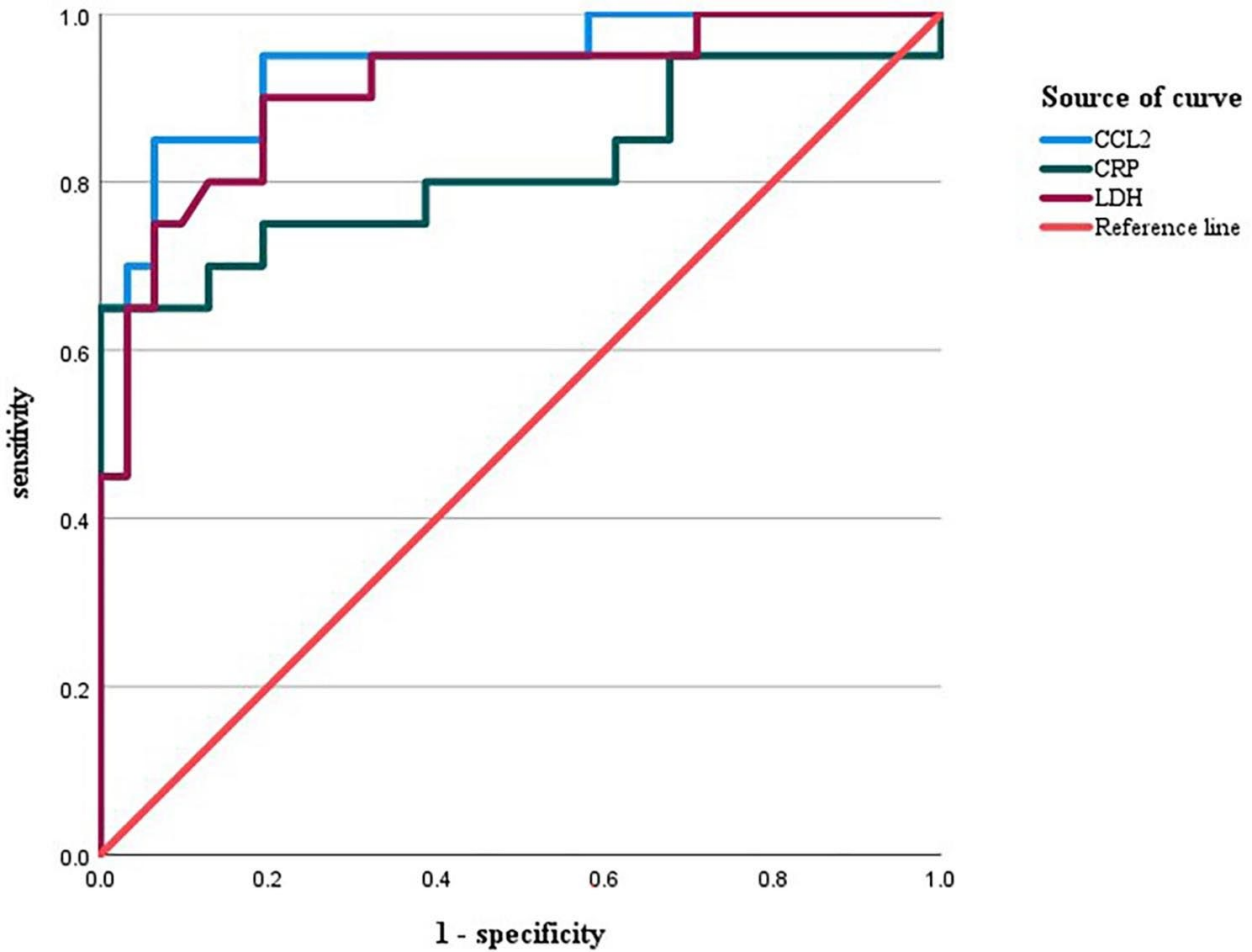
ROC curve analysis of predictive value of CCL2, serum CRP and LDH in BALF for RMPP Note: CCL2: C-C motif chemokine ligand 2; CRP: C-reactive protein; LDH: lactate dehydrogenase;BALF: bronchoalveolar lavage fluid; RMPP: refractory Mycoplasma pneumoniae pneumonia

The assessment value of CCL2 level in BALF, serum CRP and LDH for RMPP was analyzed using the ROC curve. The AUC of CCL2 predicting RMPP was 0.94, with a cut-off value of 0.645 ng/ml, a sensitivity of 85% and a specificity of 94%. The AUC of CRP was 0.816 with cutoff value of 56.2 mg/L. The AUC of LDH was 0.910 with cutoff value of 397 U/L. Table 4; Fig. 2 for details.

**Table 4:** Diagnostic value of CCL2 in BALF for RMPP

Indicators	AUC	95%CI of AUC	Cut-off value	Youden index	Sensitivity (%)	Specificity (%)
CCL2(ng/mL)	0.940	0.873 ~ 1.000	0.645	0.78	85	94
CRP(mg/L)	0.816	0.678 ~ 0.955	56.2	0.65	65	100
LDH(U/L)	0.910	0.825 ~ 0.996	397	0.71	90	81

Note: CCL2: C-C motif chemokine ligand 2; CRP: C-reactive protein; LDH: lactate dehydrogenase

#### Logistic Univariate regression analysis

With the occurrence of RMPP as the dependent variable (yes = 1, no = 0), Logistic univariate regression analysis showed RMPP was significantly associated with CCL2 in BALF, duration of fever, height, age, NE, MONO%, MONO, CRP, LDH and D-dimer (*P* < 0.05), and there was no significant association between RMPP and other variables (*P* > 0.05). To prevent excessive OR values, sextuple CCL2 analysis was used for variable CCL2 in Logistic univariate regression analysis. Results of the Logistic univariate regression analysis are shown in Table 5.

**Table 5:** Results of Logistic univariate regression analysis

Variable	b value	Standard error of b value	Wald Chi-square value	*P* value	*OR* value
Number of lung lobes invaded	0.580	0.329	3.098	0.078	1.785
Duration of fever	0.390	0.116	11.242	0.001	1.476
CCL2	6.323	2.220	8.113	0.004	557.311
Sextuple CCL2	1.054	0.370	8.113	0.004	2.869
Gender	0.211	0.582	0.132	0.716	1.235
Pre-hospital disease course	-0.056	0.044	1.680	0.195	0.945
Time from disease onset to lavage	-0.027	0.038	0.486	0.486	0.974
MP antibody	0.002	0.002	0.741	0.389	1.002
MP load in BALF	0	0	0.793	0.373	1.0
Height	2.948	1.466	4.040	0.044	19.061
Body weight	0.030	0.025	1.484	0.223	1.031
BMI	-0.052	0.098	0.274	0.600	0.950
Age	0.243	0.116	4.393	0.036	1.275
WBC	-0.054	0.065	0.697	0.404	0.947
NE%	0.166	0.049	11.387	0.001	1.180
MONO	-3.771	1.676	5.065	0.024	0.023
MONO%	-0.321	0.154	4.330	0.037	0.725
Platelet count	-0.005	0.003	3.180	0.075	0.995
CRP	0.050	0.017	8.672	0.003	1.051
LDH	0.013	0.004	9.821	0.002	1.013
D-dimer	0.773	0.237	10.608	0.001	2.167

Note: *P* < 0.05 indicates statistically significant difference; CCL2: C-C motif chemokine ligand 2; MP: Mycoplasmal pneumonia; BMI: Body Mass Index; WBC: White Blood Cell; NE%: neutrophil percentage; MONO: monocyte count; MONO%: Percentage of monocytes in white blood cells; CRP: C-reactive protein; LDH: lactate dehydrogenase

#### Predictive model I of CCL2 level on RMPP in BALF (logistic multivariate regression analysis)

**Fig. 3:**
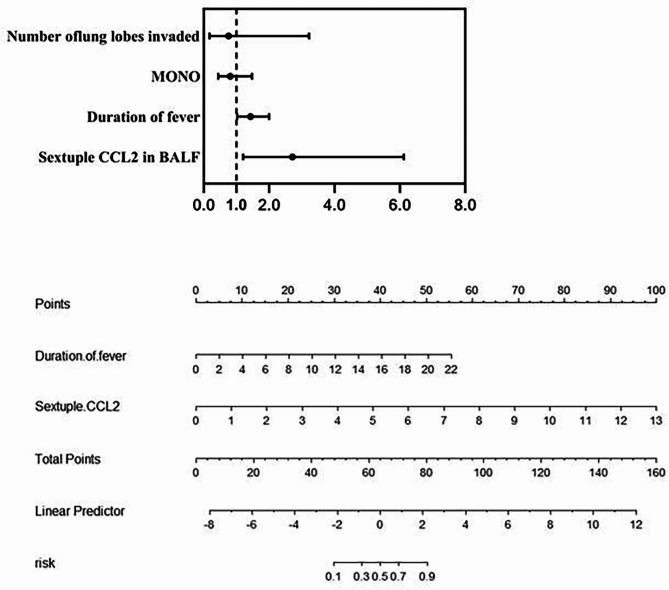
**A**. Logistic regression analysis model diagram (forest plot) Note: OR > 1 indicates that this factor is a facilitating factors for the development of RMPP, OR = 1 indicates no effect, and OR < 1 indicates a hindering factor **B**. Nomogram for predicting the risk of the RMPP occurrence Note: MONO: monocyte count; CCL2: C-C motif chemokine ligand 2; BALF: bronchoalveolar lavage fluid https://lxt134520.shinyapps.io/DynNomapp/ (Predictive model: an interactive dynamic nomogram)

The variables with statistical significance (*P* < 0.05) in the univariate analysis and those reported in the literature with significant impact on the outcome were included in the Logistic regression analysis, which showed that CCL2 in BALF and duration of fever were risk factors for the occurrence of RMPP (*P* < 0.05). The results of the Logistic multivariate regression analysis are shown in Table 6; Fig. 3A.

**Table 6:** Logistic Multivariate regression analysis

Variable	b value	Standard error of b value	Wald Chi-square value	*P* value	*OR* value
Duration of fever	0.359	0.171	4.398	0.036	1.432
Sextuple CCL2	0.999	0.415	5.802	0.016	2.716
Number of lung lobes invaded	-0.278	0.739	0.141	0.707	0.758
MONO	-0.211	0.307	0.472	0.492	0.810
Constant	-7.267	3.282	4.903	0.027	0.001

Note: *P* < 0.05 indicates statistically significant difference; CCL2: C-C motif chemokine ligand 2; MONO: monocyte count

#### Predictive model II of CCL2 level in BALF on RMPP (Nomogram)

The variables with statistically significant (P < 0.05) and those reported in the literature with significant impact on the outcome were analyzed by Logistic regression, and CCL2 and duration of fever were selected as variables. Table 7.

**Table 7:** Results of Logistic multivariate regression analysis

Variable	b value	Standard error of b value	Wald Chi-square value	*P* value	*OR* value
Duration of fever	0.340	0.163	4.336	0.037	1.405
Sextuple CCL2	1.038	0.431	5.811	0.016	2.824

Note: *P* < 0.05 indicates statistically significant difference; CCL2: C-C motif chemokine ligand 2

According to the selected variables, the nomogram of the RMPP prediction model was constructed to further visualized the risk of RMPP diagnosis. The total score is obtained by adding the score corresponding to the number of days of fever to the score corresponding to the measured value of CCL2, and the following value corresponds to the predicted risk coefficient, as shown in Fig. 3B, as shown in Fig. 3B below (Note: Based on two independent risk factors, a nomogram for predicting RMPP risk was established on the basis of the duration of fever and sextuple CCL2 to prevent excessive OR value. Specify a point for each variable by drawing a line from the corresponding variable to the dotted line. The sum of the points plotted on the total dotted line corresponds to the risk value of the RMPP risk score representing the specific risk of developing an RMPP).

### Prognosis

All the study subjects included in this study were eventually cured and discharged from hospital without death or disability.

### Discussion

In recent years, the incidence of RMPP has been increasing and the treatment is difficult. Current studies generally believe that excessive inflammatory response plays a key role in the pathogenesis of RMPP, and the early prediction of RMPP has become a focus of research. Ding Y et al. [23] have found that High Mobility Group Box 1 level is increased in the peripheral blood of children with RMPP and is a good biomarker of RMPP. Fu et al. [24] found that the level of autotaxin was significantly increased in the serum and BALF of children with RMPP, which can be used as a predictor of children RMPP. Zhang Y et al. [25] found that IL-10 and IFN-γ may be good predictors for RMPP in school-age children. Studies have proposed that cytokines can be used as new therapeutic targets to reduce the damage caused by excessive inflammatory response to the body [26]. Most of these biomarkers (including CCL2) are still under study or are not widely available in clinical practice. This study investigated the predictive value of chemokine CCL2 in BALF for RMPP in children.

In this study, by measuring CCL2 level in BALF in the study group and the control group, we found that CCL2 level in BALF was significantly higher in MPP children than that in the tracheal foreign body group, and CCL2 level in BALF in the RMPP group was significantly higher than that in the NRMPP group. This is consistent with the report of Yang M and others [22]. They measured 13 cytokines in BALF in 88 children with MPP and 26 children with foreign body inhalation (FB) with the Luminex system, and found higher CCL2 level in BALF in mild MPP children than in the FB group and higher CCL2 level in BALF in severe MPP children than in the mild MPP group, with statistically significant differences (P < 0.05). The study of Chen X et al. [27] found that CCL2 in BALF in MPP children was higher than that in bronchial foreign body group, and serum CCL2 of MPP children was not significantly different from healthy controls, suggesting that CCL2 in BALF could reflect the disease status more than serum CCL2.

In this study, by comparing clinical data and CCL 2 in BALF between the RMPP group and the NRMPP group, we found that CCL2 level in BALF, age, NE%, CRP, D-dimer, and LDH of the RMPP group were significantly higher than those of the NRMPP group, and MONO and MONO% were lower than those in the NRMPP group, with statistically significant differences (*P* < 0.05). A systematic review and Meta-analysis by Huang W et al. [28] concluded that CRP, LDH, elevated neutrophil percentage and lung consolidation were predictors of RMPP, which is consistent with our study. A study by Shen F et al. [12] found CRP, LDH and D-dimer as predictors of RMPP and developed an RMPP prediction model that can visualize and accurately quantify the risk of developing RMPP with nomograms, early identify RMPP and assist in the selection of appropriate treatment options. Plasma D-dimer (D-D) is a degradation product of cross-linked fibrin. D-D value increases when acute coagulation occurs [29] and it can be used as molecular markers of hypercoagulability. It is also a specific marker of the fibrinolytic system and can be used as one of the indicators to monitor inflammation and severe infections. The elevated plasma D-D level in MPP patients may be related to the vascular endothelial cell damage caused by the release of various inflammatory mediators from the inflammatory cells in children with MPP [30]. Huang X et al. [31] found significantly higher serum D-D level in RMPP patients, indicating excessive inflammatory response and long-lasting vascular endothelial damage in this patient population. Elevated serum D-D level can be used as an early predictor of the occurrence of RMPP and complications (pleural effusion and liver injury). In this study, we found that the median age of RMPP patients was higher than that of NRMPP patients (7.0 (6.1,9.0) vs. 5.3 (3.1,7.0)), with statistically significant difference (*P* < 0.01), which is consistent with previous reports [32]. The immune system of older children is relatively more mature and may be more likely to produce excessive immune response [33]. However, there are also different study findings. Zhai YY et al. [34] analyzed the clinical data of 20 children with RMPP and found that there was no significant difference in age between the refractory MPP group and the general MPP group. However, there were statistically significant differences in the duration of fever, CRP, ESR, LDH, PLT, WBC, D-dimer, and intrapulmonary and extrapulmonary complications (*P* < 0.05). Several studies have found a clear correlation between duration of fever and the occurrence of RMPP [12, 31], which is consistent with the results of this study.

CCL2 belongs to pro-inflammatory chemokines according to functions and belongs to monocyte/macrophage chemokines according to the cell types with different chemotactic effects. It plays an important role in chemotaxis and activation of monocytes to the site of inflammation. CCL2 is not only a specific parameter of MPP, it is usually an anti-inflammatory value. Betakova T et al. [26] summarized the data of induced cytokines and chemokines in the serum of patients infected with human and avian influenza virus, and determined that they may play a role in the pathogenesis. Chemokines CCL-2, CXCL-8, CXCL-9 and CXCL-10 are related to the pathogenicity and mortality of avian influenza virus. Khalil BA [35] and others described the role of chemokines and their receptors in the pathogenesis of COVID-19, emphasizing the most prominent chemokines related to the progression of COVID-19, including CCL2, CXCL10 and CXCL8, and their importance as potential biomarkers, which helps to determine targeted treatment options for chemokines. Lee YC et al. [36] included 42 children with MPP. Plasma samples were collected on admission and after one to two weeks of treatment with antibiotics or methylprednisolone to detect chemokine levels. M1-related chemokines (CCL2, CCL8 and CXCL10) decreased and M2-related chemokines (CCL17 and CCL22) increased in plasma of MPP children. In this study, CCL2 level in BALF was increased in children with MPP and significantly increased in children with RMPP. The assessment value of CCL2 level in BALF on RMPP was analyzed with ROC curve. The AUC of CCL2 was 0.94 and the cutoff value was 0.645 ng/ml. The AUC of CRP was 0.816 with cutoff value of 56.2 mg/L. The AUC of LDH was 0.910 with cutoff value of 397 U/L. The results showed that the AUC and cut-off value of CCL2 were significantly greater than those of CRP and LDH, and the difference was statistically significant, which indicated that CCL2 had higher accuracy in the diagnosis of RMPP. CRP and LDH in serum reflect the general state and are affected by many factors. CCL2 in bronchoalveolar lavage fluid is a local inflammatory index, which has better sensitivity and specificity, and is more stable in predicting RNMPP. Studies suggest that excessive immune response is involved in the formation of RMPP, and CCL2 is involved in the immune response. Hue Erika et al. [37] found in an animal experiment that the level of cytokines in the bronchoalveolar lavage fluid (BALF) of the affected side was significantly higher than that of the healthy side. It has a higher predictive value than CRP and LDH in predicting the occurrence of RMPP. Variables that were statistically significant in univariate analysis and those with significant impact on the outcome were included in the Logistic regression model, and CCL2 and duration of fever were selected to make a nomogram to identify the risk of RMPP. CCL2 showed good predictive value, which may be one of the biomarkers for predicting RMPP.

This study also has some limitations. BALF was not easily available due to ethical requirements, and children with bronchial foreign bodies in the same period were selected as the control group in this study. The number of patients in the control group was small due to the young age of tracheal foreign body. This study is a single-center study. Multi-center joint studies can be conducted in the future. The level of CCL2 in BALF measured in this study is lower than that reported abroad, which may be related to the difference in immune status of children in different regions. In addition, some children have been given drug treatment before the bronchoscopy lavage, which may have an impact on the measured value. In conclusion, this study has preliminarily demonstrated the good potential of CCL2 as a predictor of RMPP, and more high-quality studies are needed for subsequent validation.

### Conclusion

The present study provides the following conclusions: Elevated levels of CCL2 were observed in the BALF of children with MPP, particularly in the RMPP group compared to the NRMPP group. However, it is important to note that while the findings suggest a potential of CCL2 as a biomarker for predicting RMPP, further research is essential to validate and generalize these findings. Therefore, our study lays the foundation for future investigations into the utility of CCL2 and other predictive methods for RMPP, underscoring the importance of further research to comprehensively assess its clinical applicability.

## Data Availability

All data generated or analyzed during this study are included in this published article.

## Abbreviations

CCL2: C-C motif chemokine ligand 2
BALF: Bronchoalveolar lavage fluid
MPP: Mycoplasma pneumoniae pneumonia
AUC: Area under the curve
CAP: Community-acquired pneumonia
MP: Mycoplasma
RMPP: Refractory mycoplasma pneumoniae pneumonia
MRMP: Macrolide-resistant Mycoplasma pneumoniae
BALF: Bronchoalveolar lavage fluid
NRMPP: Non-refractory mycoplasma pneumoniae pneumonia
IQR: Interquartile range
ROC: Receiver operating characteristic
D-D: D-dimer
